# Identification of the Relationship between Oil Body Morphology and Oil Content by Microstructure Comparison Combining with QTL Analysis in *Brassica napus*

**DOI:** 10.3389/fpls.2016.01989

**Published:** 2017-01-06

**Authors:** Jianwei Gu, Hongbo Chao, Hao Wang, Yonghong Li, Dianrong Li, Jun Xiang, Jianping Gan, Guangyuan Lu, Xuekun Zhang, Yan Long, Maoteng Li

**Affiliations:** ^1^Department of Biotechnology, College of Life Science and Technology, Huazhong University of Science and TechnologyWuhan, China; ^2^Hubei Collaborative Innovation Center for the Characteristic Resources Exploitation of Dabie Mountains, Huanggang Normal UniversityHuanggang, China; ^3^Hybrid Rapeseed Research Center of Shaanxi Province, Shaanxi Rapeseed Branch of National Centre for Oil Crops Genetic ImprovementYangling, China; ^4^Oil Crops Research Institute, Chinese Academy of Agricultural SciencesWuhan, China; ^5^Institute of Biotechnology, Chinese Academy of Agricultural SciencesBeijing, China

**Keywords:** oil body, *Brassica napus*, oil content, fatty acids, QTL, map alignment

## Abstract

Oil bodies (OBs) are relatively simple but very important organelles comprising a matrix of triacylglycerol (TAG) surrounded by a phospholipid monolayer embedded and covered with unique proteins. The OB structure in *Brassica napus* with different oil content and the relationship between the oil content and the OB structure needs to be better understood. In this paper, the characteristics of OBs in the embryo of a series of *B. napus* materials with different oil content ranging from 34% to over 60% were studied. The results indicated that the OB size was significantly positively correlated with the oil content but was significantly negatively correlated with the glucosinolates and the protein content. Many genes associated with TAG synthesis, OB-membrane proteins, and the cell progress regulatory pathway were identified in the confidence interval of co-located QTLs for oil content, fatty acid (FA) compositions, and protein content. Our results suggested that the morphology of OBs might be directly controlled by the genes associated with OB-membrane proteins and indirectly controlled by the genes associated with TAG synthesis and cell progress regulatory pathway.

## Introduction

Rapeseed (*Brassica napus*, AACC, 2*n* = 38) is the second important oilseed crop in the world which occupies about 13–16% of the world vegetable oil production (Hajduch et al., 2006; Wang and Yin, 2014). The demand of vegetable oils for food, fuel (bio-diesel), and bioproduct applications has rapidly increased in the past years (Vigeolas et al., 2007; Wang and Yin, 2014).

Some researches revealed that the oil content of the existing *B. napus* germplasm varied from 26 to 51% (Fu, 2004); however, the oil content in the majority of the commercial materials was ~43% (Liu et al., 2008). So, development of the improved germplasm with higher oil is the most important breeding goals in *B. napus* at present. Significant genetic variations for oil content (ranging from 50 to 60%) were observed in both spring and winter gene pools of *B. napus* (Olsson, 1960; McVetty et al., 2007; Fu et al., 2008; Liu et al., 2008; Li et al., 2011; Hu et al., 2013). The seeds accumulated lipids are generally stored as TAGs in small, rounded, and discrete intracellular organelles called OBs (Huang, 1992; Tzen et al., 1993; Siloto et al., 2006; Shimada and Hara-Nishimura, 2010; Zienkiewicz et al., 2010). Seed OBs are simple organelles comprising a matrix of TAG surrounded by a phospholipid monolayer embedded and covered with OB-membrane proteins called oleosins, caleosins, and steroleosins (Tzen et al., 1990; Ting et al., 1996; Siloto et al., 2006; Bhatla et al., 2010; Hu et al., 2013). In rapeseed, OBs could resist coalescence and remain as small individual units during final stages of seed maturation, which mainly existed in cotyledons and the embryonic axis (Siloto et al., 2006). It has been generally accepted that the physiological significance of maintaining the OBs as small, individual entities is to provide ample surface areas for the attachment of lipase during germination (Vance and Huang, 1987; Wang and Huang, 1987; Tzen et al., 1993).

Generally, the diameter of an OB is ranged from 0.5 to 2.5 μm in *B. napus* (Tzen et al., 1993; Murphy, 2001; Mantese et al., 2006). Hu et al. (2009) reported that the low accumulation of oleosins resulted in the formation of unusually large OBs in low oil content materials and showed high correlation with low oil content in *B. napus*. Oleosin is the most important structural protein of OBs, and it could maintain the OBs as small single units and prevent them of coalescence during seed desiccation (Ross et al., 1993; Tzen et al., 1993; Ting et al., 1996; Miquel et al., 2014). Oleosin suppression resulted in an aberrant phenotype of embryo cells that contained unusually large OBs which were not normally observed in seeds (Siloto et al., 2006). Besides the consequences in OB morphology, the suppression of the major oleosin (OLEO1) caused the disruption of storage organelles, altering accumulation of lipids and proteins, increasing in C20:1 at the expense of C18:1 in TAGs (Siloto et al., 2006). The same phenomena was also observed by Lu et al. (2006), who showed changes in TAG composition as a function of oleosin over-expression in *Arabidopsis*. Shimada et al. (2008) reported an inverse relationship between OB size and the content of total oleosin in *Arabidopsis*. Hu et al. (2013) reported the seed ultrastructure of an ultrahigh oil content rapeseed material with 64.8% of oil content, and indicated that this material had a higher OB organelle to cell area ratio than that of the materials with low oil content (Hu et al., 2013). All these results implied that the morphology of OBs was correlated with the seed oil content and FA compositions, in which OB-membrane proteins might play important roles. But, the OB characteristics in *B. napus* materials with ultrahigh oil content and the relationship of OB morphology with the oil content and FA compositions remain in need to be better understood.

Several proposals were made for OB formation (Murphy, 2001; Yang et al., 2012). The OBs started from a site of the ER specialized in TAG synthesis and would then expand by recruiting enzymes of this synthetic pathway (Murphy, 2001; McFie et al., 2011; Wilfling et al., 2013). Then, OBs would remain tethered to ER, filled with neutral lipids until an optimal size was reached, allowing their release into the cytosol (Kuerschner et al., 2008). Finally, small OBs could form into larger ones by fusion or coalescence of each other (Miquel et al., 2014). These different mechanisms might depend on the type of lipid droplets (LDs), the cell type and the neutral lipids accumulated (Cheng et al., 2009; Thiam et al., 2013). The OB morphology might be determined by a highly coordinated process that involves carbon metabolism, FA synthesis, TAG synthesis pathways, and even cell differentiation pathways, so the seed OB morphology should be affected by many factors. Wilfling et al. (2013) confirmed that larger LDs containing isoenzymes for each step of TAG synthesis in *Drosophila* and endoplasmic reticulum (ER)-to-LD targeting GPAT4 and other LD-localized TAG synthesis isozymes were required for LD growth. Moreover, it was confirmed that over-expression of *BnDGAT2* caused the alteration of the FAs profile and the formation of a large number of lipid globules in the transformed alga (Ahmad et al., 2015). These results indicated that the roles of these genes associated with the TAG synthesis pathway in oil storage organs in plants might be similar to those in animals. Beside these TAG synthesis related genes, reverse genetic analysis revealed that oleosins function as a size-regulator of OBs (Lu et al., 2006; Siloto et al., 2006). Caleosin might directly involve in membrane and OB fusion (Næsted et al., 2000). Steroleosin may be involved in the activation of sterol signal transduction that regulates specialized biological functions related to the formation or mobilization of OBs during seed maturation or germination (Lin and Tzen, 2004). Less abundant proteins in OBs may also play roles in OB formation, for instance, phospholipase D1 (*PLD1*), and extracellular signal-regulated kinase 2 (*ERK2*) are known to regulate cytosolic OB formation in animal cells (Andersson et al., 2006). *Growth-regulating factor* (*GRF*) is a transcription factor which has the function in female reproduction development and ovule formation in *Arabidopsis* (Wynn et al., 2011). It has been revealed that the over-expression of the *BnGRF2a* in *Arabidopsis* could result in an increase of seed oil production. Furthermore, transcriptome analyses indicated that some genes associated with cell proliferation, photosynthesis, oil synthesis, and storage processes were up-regulated by the over-expression of *BnGRF2a*, such as *KCS16, GPAT*, and oleosin proteins (Liu et al., 2012). These results indicated that the OB formation might be related to the genes associated with carbon metabolism, FA synthesis, TAG synthesis, cell division, cell growth, and cell proliferation.

Mapping quantitative trait loci (QTL) onto linkage maps using different segregating populations is a powerful genetic approach to dissect complex metabolism fact and cell functions. Fourty-six distinct QTL regions that control seed-oil content were identified on 16 of the 19 linkage groups of *B. napus* by using a high-density TN genetic map (Jiang et al., 2014). A large and new double haploid (DH) population containing 348 lines was obtained between “KenC-8” and “N53-2” by Wang et al. (2013) and a total of 63 identified QTLs explaining 2.64–17.88% of oil content variation were identified. Moreover, important candidate genes could be easily identified by mapping in the corresponding QTL regions and already used by several researchers in *B. napus* (Long et al., 2007; Gan et al., 2013; Wang et al., 2015), *Zea mays* (Dell'Acqua et al., 2015), and *Arabidopsis* (Oakley et al., 2014). Gan et al. (2013) compared the candidate genes in the QTL-CI for oil and protein content with the genes that corresponded to different expression proteins (DEPs). One hundred and seventeen candidate genes that corresponded to DEPs showed a good corresponding relationship with QTL-CIs for oil content, indicating that these DEPs might be involved in oil or protein formation. Wang et al. (2015) investigated the genetic basis of FA bio-synthesis in *B. napus* and constructed a potential regulatory pathway controlling the FA via QTL analysis using a doubled haploid (DH) population with 202 materials and mapping analysis *in silico*.

To establish the relationship among the OB morphology, oil content, and FA compositions, the characteristics of OBs in *B. napus* materials varying in oil content from 34.54% to over 60% were compared. A potential regulatory pathway controlling the relationship between the OB morphology with the oil content and FA compositions was constructed. This study would present a comprehensive insight for the relationship between OB characteristics with oil content and FA compositions in *B. napus* and would be helpful for breeding new materials with high oil content.

## Materials and methods

### Plant materials

Twenty-four *B. napus* materials which exhibited a wide range of oil content in mature seeds were used in this study (Table 1). The materials were divided into four groups based on our observation and previous paper (Gunstone et al., 2007): the ultrahigh oil group (UO, oil content over 55%), the high oil content group (HO, oil content of 50–55%), the medium oil content group (MO, oil content of 40–50%), and the low oil content group (LO, oil content of 30–40%). The ANOVA analysis by EXCEL showed the significant differences among LO, MO, HO, and UO group in oil content (data not shown). All these materials were provided by Shaanxi Hybrid Rapeseed Research Center and Huazhong University of Science and Technology. All materials were grown at DaLi in Shaanxi Province and WuHan of Hubei Province, which belonged to winter and semi winter rapeseed planting areas (from September to May of the next year). The planting followed a randomized complete-block design with three replicates. Each plot was 3.0 m^2^ with 30 plants in all microenvironments with 40 cm between rows and 25 cm between individual plants (Wang et al., 2013). All materials are sown on the same day (September 20 in DaLi and October 5 in WuHan) and the seeds were harvested when over 75% of the pods in the plot become from green to yellow color.

**Table 1:** **Average OB size and 10 biggest OBs of each *B. napus* cultivars**.

**No**.	**Group**	**Materials**	**Oil content (%)**	**Average OBs size for group (μm^2^)**	**Average OBs size (μm^2^)**	**Cross-sectional area (μm^2^)**									
1	LO	13123F	34.54 ± 2.82	0.22 ± 0.02	0.31	1.28	1.25	1.24	1.22	1.21	1.21	1.19	1.19	1.14	1.13
2		12QT329	36.56 ± 2		0.14	1.15	1.08	1.08	1.04	0.95	0.87	0.78	0.78	0.76	0.75
3		10QT0073	39.16 ± 1.76		0.18	77.25	69.38	42.92	42.48	36.8	36.8	30.3	2.65	2.54	1.46
4		12QT103	39.95 ± 2.20		0.24	6.61	5.25	5.25	4.82	4.82	4.11	4.11	2.59	2.59	2.57
5	MO	13171	40.88 ± 2.27	0.19 ± 0.01	0.39	5.85	4.31	3.79	2.66	2.23	2.06	2.02	2	1.75	1.62
6		11DH627	41.27 ± 1.39		0.11	9.48	2.85	2.06	1.67	1.63	1.59	1.57	1.56	1.56	1.56
7		09SN620	41.51 ± 1.28		0.28	2.16	1.88	1.86	1.67	1.46	1.43	1.35	1.12	1.11	1.04
8		09QTL1	41.61 ± 2.31		0.3	1.96	1.19	1.16	0.95	0.85	0.85	0.83	0.79	0.74	0.73
9		13042F	44.79 ± 0.72		0.24	8.54	7.53	7.13	6.43	6.37	5.8	5.49	4.14	3.6	3.58
10		12QT127	45.01 ± 0.25		0.2	2.15	1.03	0.97	0.96	0.9	0.89	0.81	0.8	0.79	0.78
11		09QT68	46.25 ± 3.99		0.19	3.21	2.18	1.06	1.06	1.03	1.03	1	0.97	0.91	0.91
12		13066F	48.5 ± 1.03		0.16	2	1.96	1.94	1.93	1.57	1.5	1.33	1.28	1.25	1.22
13		09QT102	48.57 ± 2.57		0.18	6.86	6.47	6.45	5.97	5.02	4.62	3.7	1.77	1.6	1.21
14	HO	11DH623	50.68 ± 2.30	0.20 ± 0.02	0.43	18.15	13.73	10.98	10	8.72	7.2	6.35	5.89	5.85	5.63
15		14309	52.66 ± 2.37		0.21	6.96	5.81	4.73	3.2	3.05	2.95	2.7	2.7	2.43	2.36
16		14313	52.75 ± 2.90		0.76	11.72	7.65	7.6	6.39	4.58	4.21	3.33	3.16	3.08	2.75
17		07WHQT11	54.12 ± 3		0.14	2.26	1.06	0.56	0.5	0.46	0.45	0.44	0.43	0.41	0.41
18	UO	TGY8	55.04 ± 1.02	0.57 ± 0.05	0.92	15.16	12.34	11.51	10.12	8.3	7.75	7.67	7.31	7.2	6.81
19		TGY10	58.57 ± 1.22		0.48	20.23	16.72	12.37	10.3	9.27	8.68	8.15	8.03	6.89	6.87
20		TGY18	58.89 ± 0.94		0.65	3.43	2.14	1.85	1.85	1.82	1.8	1.75	1.69	1.66	1.66
21		TGY6	59.01 ± 3.28		0.52	8.75	5.27	5.14	5.08	4.83	4.72	4.32	4.3	4.27	3.82
22		TGY14	59.86 ± 1.33		0.22	22.59	11.38	5.6	3.42	3.08	3	2.97	2.85	2.81	2.79
23		TGY16	60.16 ± 3.27		0.16	9.22	7.17	6.81	6.45	4.66	2.5	2.21	1.22	1.2	1.16
24		TGY17	60.61 ± 2.22		0.37	7.35	5.31	4.06	3.85	3.6	3.53	3.46	3.19	3.1	2.98

### Oil content, protein content, and FA compositions analysis of *B. napus* seeds

Open-pollinated mature seeds were obtained from randomized growth trial containing 30 plants for each material. The FA compositions in seeds were determined by near-infrared reflectance spectroscopy (NIRS; Foss NIRSystems Inc., USA) using standard methods with three replicates (Mika et al., 2003). Seed oil content and protein content was determined by using nuclear magnetic resonance (NMR) (mq-20, Bruker, Germany) with at least five biological replicates according to Burns et al. (2003) with modifications. The glucosinolates content in seeds was determined by using UPLC (Waters ACQUITY, USA) as described by Gratacós-Cubarsí et al. (2010) with three replicates.

### Transmission electron microscopy analysis of embryos of *B. napus* seeds

Cotyledons from mature *B. napus* seeds were isolated and stained according to the method of Siloto et al. (2006). The central part of the semicircular cotyledons was fixed with 2.5% glutaraldehyde in a 0.1 M phosphate buffer (pH 6.8) for 24 h, and postfixed with a 1% osmium tetroxide solution for an additional 2 h. After dehydration by using the acetone series (20, 50, 70, 90, and 3 × 100% v/v), the cotyledons were filtrated and subsequently embedded in 100% (w/v) EPON-812 epoxy resin (Sigma-Aldrich, America). Ultrathin sections (0.7 μm) were prepared by using a diamond knife on a UC6 Ultratome (Leica, Germany) onto copper grids, and stained with uranyl acetate and lead citrate. Images were acquired by using an H-7650 transmission electron microscope (TEM) (Hitachi, Japan).

### Measurement and statistical analysis of the OBs and other related traits

The number of OBs and protein storage vacuole (PSV), the OBs and PSVs size, the cell wall thickness, the cross-sectional area of cotyledon cells were measured by the Image-Pro Plus (IPP) software (Hu et al., 2013). In order to understand the relationship between OB morphology and oil content, the histogram distribution of OBs size in these materials was analyzed by Microsoft Excel 2010. As most of the OBs size was smaller than 2 μm^2^, the distributed interval was divided into 0–0.4, 0.4–0.8, 0.8–1.2, 1.2–1.6, 1.6–2.0, and over 2.0 μm^2^. The *T*-test was used to determine the significance of differences between means.

### Hierarchical cluster and correlation analysis

The Pearson correlation coefficient between OB morphology and other traits were analyzed with IBM SPSS statistics 20 (Tables 2, 3). We also calculated the frequency distribution of all OBs according to the OBs size. The distribution range was set to 0–2 μm^2^, and the distribution pitch was of 0.2 μm^2^. The frequency distribution data was used for subsequent hierarchical clustering by PermutMatrixEN software. Hierarchical clustering was performed with McQuitty's method (WPGMA). Other statistical data (i.e., average OBs size, average OBs coverage, average PSVs size, average PSVs coverage, average cell wall thickness (Table 3), and so on) were also used for hierarchical clustering after being processed as the same way.

**Table 2:** **Oil content, protein content, and major FA compositions of *B. napus* seeds in different materials**.

**No**.	**Group**	**Materials**	**Oil content (%)**	**Protein content (%)**	**Oleic acid (%)**	**Linoleic acid (%)**	**Linolenic acid (%)**	**Erucic acid (%)**	**Saturated fatty acid (%)**	**Glucosinolates (μmol/g)**
1	LO	13123F	34.54 ± 2.82	29.14 ± 1.62	51.13 ± 2.56	25.16 ± 1.38	12.64 ± 0.69	0.00	6.2 ± 0.28	98.99 ± 5.5
2		12QT329	36.56 ± 2	29.94 ± 1.66	43.78 ± 2.35	16.66 ± 0.93	9.04 ± 0.5	19.03 ± 1.05	4.77 ± 0.41	100.82 ± 5.6
3		10QT0073	39.16 ± 1.76	27.43 ± 1.52	18.23 ± 0.96	14.62 ± 0.81	9.28 ± 0.52	35.98 ± 1.61	5.91 ± 0.32	58.83 ± 3.27
4		12QT103	39.95 ± 2.20	28.63 ± 1.59	45.86 ± 2.55	15.98 ± 0.73	7.83 ± 0.43	16.57 ± 0.75	4.36 ± 0.24	84.27 ± 4.58
5	MO	13171	40.88 ± 2.27	28.18 ± 1.39	61.62 ± 3.05	23.44 ± 1.3	9.63 ± 0.51	0.00	6.62 ± 0.37	16.06 ± 0.85
6		11DH627	41.27 ± 1.39	27.26 ± 1.51	58.4 ± 2.65	20.61 ± 1.15	9.8 ± 0.52	1.99 ± 0.09	5.81 ± 0.32	15.33 ± 0.81
7		09SN620	41.51 ± 1.28	28.14 ± 1.56	49.85 ± 2.25	24.84 ± 1.24	10.8 ± 0.6	0.78 ± 0.04	6.33 ± 0.34	15.01 ± 0.82
8		09QTL1	41.61 ± 2.31	25.13 ± 1.37	61.88 ± 3.4	20.64 ± 1.11	9.3 ± 0.46	1.79 ± 0.09	6.53 ± 0.12	82.73 ± 4.22
9		13042F	44.79 ± 0.72	24.86 ± 1.32	18.5 ± 0.94	12.95 ± 0.68	8.72 ± 0.46	38.04 ± 1.89	4.02 ± 0.21	66.51 ± 3.65
10		12QT127	45.01 ± 0.25	29.35 ± 1.54	8.52 ± 0.47	13.63 ± 0.76	10.88 ± 0.6	43.57 ± 1.98	3.7 ± 0.21	164.99 ± 7.37
11		09QT68	46.25 ± 3.99	22.77 ± 1.25	40.42 ± 1.8	14.02 ± 0.69	8.12 ± 0.4	19.71 ± 0.89	5.77 ± 0.32	98.02 ± 4.86
12		13066F	48.5 ± 1.03	22.55 ± 1.02	56.65 ± 2.57	24.6 ± 1.12	10.79 ± 0.53	0.00	6.4 ± 0.45	27.43 ± 1.52
13		09QT102	48.57 ± 2.57	22.53 ± 1.01	25.66 ± 1.43	11.54 ± 0.52	8.81 ± 0.49	39.27 ± 2.18	5.2 ± 0.28	63.51 ± 3.52
14	HO	11DH623	50.68 ± 2.30	23.43 ± 1.3	26.25 ± 1.46	11.93 ± 0.66	7.57 ± 0.42	36.96 ± 2.05	5.64 ± 0.3	24.2 ± 1.32
15		14309	52.66 ± 2.37	19.45 ± 1.08	24.87 ± 1.32	15.6 ± 0.82	6.73 ± 0.37	32.07 ± 1.78	4.53 ± 0.25	66.41 ± 3.52
16		14313	52.75 ± 2.90	20.39 ± 1.11	17.97 ± 0.94	14.63 ± 0.81	7.89 ± 0.42	35.56 ± 1.98	4.5 ± 0.12	82.1 ± 4.56
17		07WHQT11	54.12 ± 3	20.27 ± 1.13	18.95 ± 0.96	7.11 ± 0.35	9.15 ± 0.51	56.24 ± 3.06	4.66 ± 0.21	65.37 ± 3.63
18	UO	TGY8	55.04 ± 1.02	11.45 ± 0.57	53.38 ± 2.93	18.14 ± 1	13.65 ± 0.68	0.82 ± 0.04	5.89 ± 0.2	21.54 ± 1.17
19		TGY10	58.57 ± 1.22	11.26 ± 0.63	17.09 ± 0.94	8.08 ± 0.45	10.93 ± 0.61	42.89 ± 2.39	4.57 ± 0.25	60 ± 3.18
20		TGY18	58.89 ± 0.94	11.32 ± 0.63	60.46 ± 3.35	14.45 ± 0.79	11.33 ± 0.63	1.72 ± 0.09	5.29 ± 0.28	26.01 ± 1.43
21		TGY6	59.01 ± 3.28	12.57 ± 0.68	6.28 ± 0.34	6.36 ± 0.35	11.56 ± 0.64	51.65 ± 2.84	4.49 ± 0.25	14.1 ± 0.77
22		TGY14	59.86 ± 1.33	10.74 ± 0.57	31.68 ± 1.68	9.57 ± 0.53	13.62 ± 0.75	26.47 ± 1.35	4.61 ± 0.14	54.45 ± 2.87
23		TGY16	60.16 ± 3.27	13.03 ± 0.72	11.21 ± 0.56	5.67 ± 0.31	12.33 ± 0.63	46.82 ± 2.57	4.36 ± 0.23	45.29 ± 2.52
24		TGY17	60.61 ± 2.22	10.59 ± 0.52	18.6 ± 1	5.97 ± 0.32	11.57 ± 0.64	41.29 ± 2.22	4.53 ± 0.1	20.87 ± 1.03

*The error in table represents the stand error calculated by EXCEL*.

**Table 3:** **Correlation coefficients of seed traits of 24 *B. napus* materials**.

	**Oleic acid**	**Linoleic acid**	**Linolenic acid**	**Erucic acid**	**Saturated fatty acid**	**Glucosinolates**	**Protein content**	**Oil content**	**Average PSV size**	**Average OB size**	**PSV coverage**	**OB coverage**	**Cell wall thickness**	**Cell size**
Oleic acid	1													
Linoleic acid	0.799^**^	1												
Linolenic acid	0.083	−0.05	1											
Erucic acid	−0.962^**^	−0.882^**^	−0.164	1										
Saturated fatty acid	0.758^**^	0.738^**^	0.069	−0.767^**^	1									
Glucosinolates	−0.242	−0.019	−0.236	0.226	−0.382	1								
Protein	0.293	0.630^**^	−0.496^*^	−0.319	0.306	0.420^*^	1							
Oil content	−0.450^*^	−0.723^**^	0.349	0.483^*^	−0.439^*^	−0.365	−0.947^**^	1						
Average PSV size	0.075	−0.126	0.244	−0.061	−0.254	0.145	−0.279	0.282	1					
Average OB size	−0.164	−0.316	0.31	0.154	−0.046	−0.411^*^	−0.531^**^	0.474^*^	0.171	1				
PSV coverage	−0.027	0.034	−0.174	0.078	−0.236	0.621^**^	0.422^*^	−0.38	0.36	−0.491^*^	1			
OB coverage	−0.099	−0.169	0.244	0.041	0.12	−0.607^**^	−0.518^**^	0.480^*^	−0.177	0.644^**^	−0.938^**^	1		
Cell wall thickness	−0.189	−0.032	−0.162	0.137	0.073	0.103	0.192	−0.264	−0.183	−0.07	−0.038	0.038	1	
Cell size	−0.258	−0.313	0.141	0.26	−0.006	−0.084	−0.167	0.053	−0.161	0.171	−0.011	−0.007	0.497^*^	1

* Significant at p = 0.05;

** *Significant at p = 0.01*.

### QTL mapping for oil and protein content and map alignment between *B. napus* with *Arabidopsis*

The TN DH population, with 202 DH materials derived from the cross Tapidor × Ningyou 7 constructed by Qiu et al. (2006) was used for QTL analysis of oil, protein, and FA compositions. The relative data of QTL for oil and protein content and FA compositions were obtained by Gan et al. (2013) and Wang et al. (2015). The segregating DH population named as KN also was used in this experiment. The KN DH population was derived from a cross between the parental lines KenC8 with low oil content and N53-2 with high oil content), which was firstly constructed by Wang et al. (2013). Comparative analysis between *Arabidopsis* and TN/KN linkage groups was according to the method of Long et al. (2007). A gene was considered to be candidate gene associated with the QTL when it is located within the CI.

### Genetic interaction analysis between candidate genes

The network was analyzed using String (http://string-db.org/) and visualized by NetworkAnalyzer (a plug of Cytoscape_V3.2.0). The neighborhood connectivity, the betweenness centrality, and the edge betweenness were calculated by NetworkAnalyzer. The combined score was calculated in the multiple names module of String and the organism was set as *Arabidopsis thaliana*. The neighborhood connectivity, the betweenness centrality, the edge betweenness and the combined score were mapped as node color, node size, edge size, and edge color, respectively. The network was laid out by using group attributes layout.

### Construction of potential interaction pathways of OB morphology, cell progress, oil content, and FA compositions in *B. napus*

The Kyoto Encyclopedia of Genes and Genomes (KEGG) database (http://www.kegg.jp/) was applied to identify pathways in which these candidate genes were involved (Ogata et al., 1999). Potential interaction pathways of OB morphology, oil content, and FA compositions in *B. napus* were inferred based on the pathways of FA synthesis, TAG synthesis, and the controlling of cell progress (cell growth, cell division, and cell proliferation) within *Arabidopsis* and *B. napus* (Cernac and Benning, 2004; Chia et al., 2005; Sidorov and Tsydendambaev, 2014; Wang et al., 2015) and also by using of the gene interaction analysis.

### Quantitative real-time polymerase chain reaction (qRT-PCR) analysis for key genes in the potential interaction pathways

qRT-PCR was conducted for 10 key genes that selected in the potential interaction pathways. RNAs were extracted and quantified from different developmental stages of seed in *B. napus* [1, 2, 3, 4, 6, and 7 week after flower (WAF)] followed by the user's manual of RNA prep Pure Plant Kit (TOYOBO, DP441). Expression analysis was performed with SYBR premix EX TaqTM kit (TaKaRa, Japan) on ABI 7900HT Fast Real-Time PCR System (Applied Biosystems, Framingham, USA). For each reaction, three technical replicates were conducted. The primers of the target genes and reference gene were listed in Table S1.

## Results

### Oil content, protein content, and FA compositions analysis in different *B. napus* materials

To investigate the correlation among the morphology of OB, oil content, FA compositions, and protein content in different rapeseed materials with great difference in oil content, seven UO materials, four HO materials, nine MO materials, and four LO materials were chosen (Table 1). The biggest oil content difference among these materials exceeded 25% (TGY17 and 13123F) and stable in difference was seen (Table 1 and Table S2). Further analysis of mature seeds revealed that protein were comprised only in 10.59–13.03% of the total storage reserves in UO materials, 19.45 to 23.43% in HO materials, 22.53 to 29.35% in MO materials, and 28.63 to 29.94% in LO materials (Table 2).

As shown in Table 2, there were three types with a high, medium or low erucic acid (C22:1) content. The highest C22:1 content was over 35%, but <2% in the low C22:1 materials. Besides C22:1, the content of oleic acid (C18:1) were 49–61% in the eight materials with low C22:1 content, and 6–25% in the high C22:1 content materials. The variation of linoleic acid (C18:2) and saturated fatty acid were similar to C18:1, but the linolenic acid (C18:3) and glucosinolates content changed a lot in different rapeseed materials with no regularity.

The correlation coefficients among oil content, protein content, and FA compositions were also calculated (Table 3). The results revealed that there was a significant negative relationship between oil and protein content in mature seeds, with a correlation coefficient of −0.95 (*p* < 0.01). It was showed that C18:1, C18:2, and saturated FA were significantly negatively correlated with C22:1, and the coefficients were of −0.962 (*p* < 0.01), −0.882 (*p* < 0.01), and −0.767 (*p* < 0.01), respectively. Meanwhile, C18:1, C18:2, and saturated FA were significantly negatively correlated with oil content, with correlation coefficients of −0.45 (*p* < 0.05), −0.723 (*p* < 0.01), and −0.439 (*p* < 0.05), respectively. Consistent with that, C18:2 and saturated FA were significantly positively correlated with C18:1, with coefficients of 0.799 (*p* < 0.01) and 0.758 (*p* < 0.01), respectively. A positive correlation was also observed between the oil content and C22:1, with a correlation coefficient of 0.483 (*p* < 0.05). Our results indicated that some FA compositions correlated with each other in different oil content rapeseed materials. Moreover, long chain FA was significantly positively correlated with oil content but short chain FA was on the contrary.

### Comparative analysis of OBs morphology and related traits in different rapeseed materials

**Figure 1:**
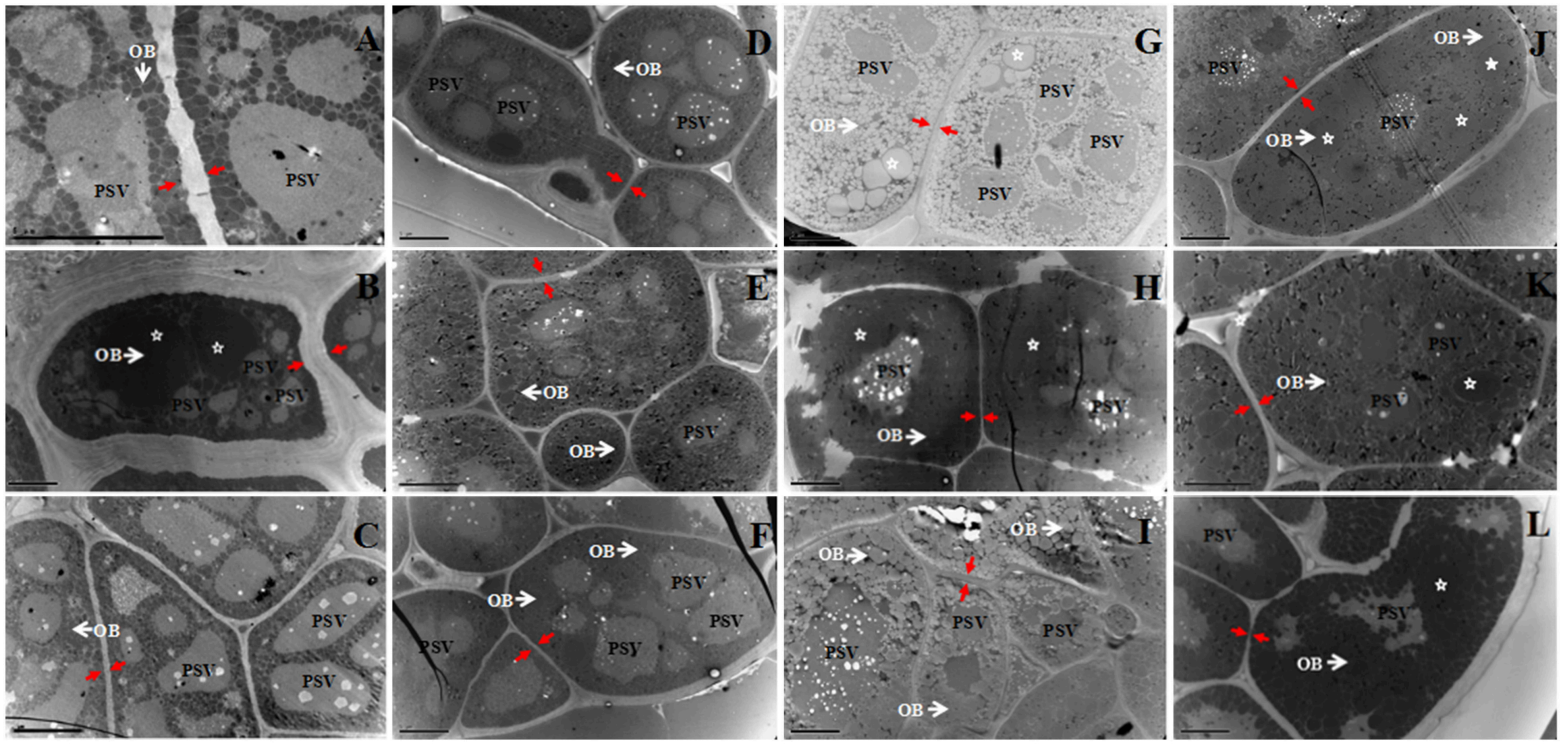
**TEM analysis of the cotyledon cells of 12 rapeseed materials with different oil content**. **(A)**, 12QT329; **(B)**, 10QT0073; **(C)**, 13123F; **(D)**, 12QT127; **(E)**, 09QT68; **(F)**, 09QTL1; **(G)**, 14313; **(H)**, 11DH623; **(I)**, 14309; **(J)**, TGY8; **(K)**, TGY10; **(L)**, TGY14; White arrow and OB, oil body; PSV, protein body; Red arrow, cell wall; Star, unusually large OB; Bar, 5 μm.

**Figure 2:**
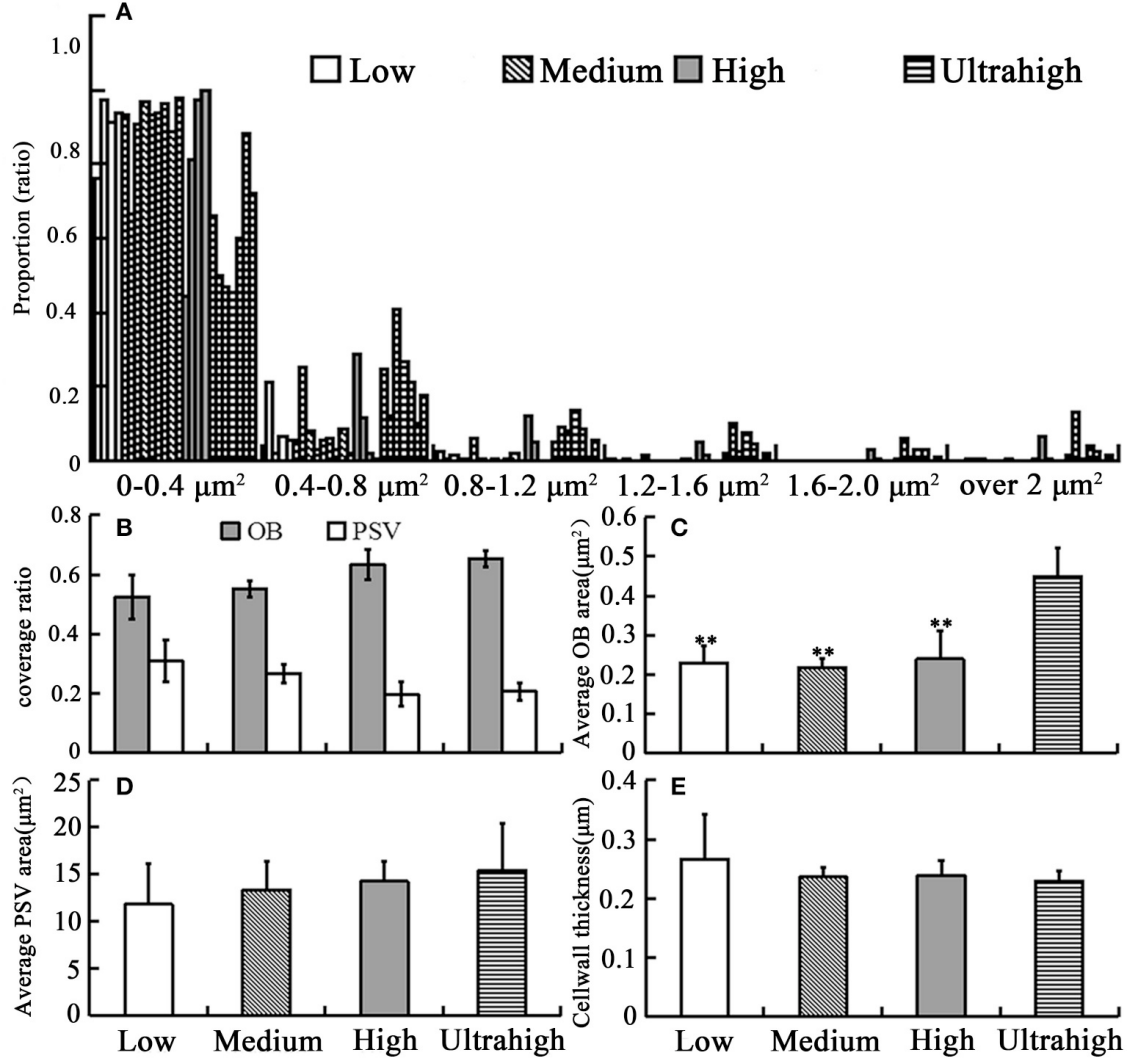
**Statistical analysis results of the related morphology traits in the cotyledon cells of several *B. napus* materials with large difference in oil content**. **(A)** histogram distribution of OB size of all materials; **(B)** the coverage of OB and PSV; **(C)** the average cross-sectional area of OB; **(D)** the average size of PSV; **(E)** cell wall thickness. At least three repeats for each set of data. Bar means SE. ^**^Significant difference (*p* < 0.01).

To make clear the relationship between OBs morphology and oil content in mature seeds, we conducted TEM analysis of OBs and other characteristics of embryonic cell among these *B. napus* materials. In mature dry seeds, the cytoplasm of cotyledon cells was completely filled with OBs and PSVs (Figure 1). The OB organelles were comprised in 67% of the total cell area in the UO group, but only 57% in the HO group, 54% in the MO group, and 52% in the LO group (Figure 2). Almost all OBs were spherical in shape, ranging in size from 0.01 to 2 μm^2^ with the majority of OBs < 0.8 μm^2^. As shown in Figure 2A, while the materials with UO were observed, only 44–72% of OBs were ranged from 0–0.4 μm^2^, 12–40% of OBs were ranged from 0.4 to 0.8 μm^2^, 5–13% were ranged from 0.8 to 1.2 μm^2^, 1–9% were ranged from 1.2 to 1.6 μm^2^, 0.1–5% were ranged from 1.2 to 1.6 μm^2^, 0.1–5% were ranged from 1.6 to 2.0 μm^2^, and 0.1–12% over 2 μm^2^. While in other materials, 76–99% of OBs were ranged from 0 to 0.4 μm^2^ and 0.3 to 21% were ranged from 0.4 to 0.8 μm^2^. That means only no more than 3% of OBs was bigger than 0.8 μm^2^ in the LO, MO, and HO materials, which was clearly lower than in UO materials (Figure 2A). Consistent with that, the average size of OBs in UO group was clearly bigger than in other groups, while the average size of OBs in the other three groups had no obvious differences (Figure 2C; excluded 11DH623 and TGY10, with an average OB size of 0.76 and 0.92 μm^2^, Table S3).

The 10 biggest OBs of each material were shown in the Table 1, the result revealed that few OBs were bigger than 2 μm^2^ in cross-sectional. An interesting phenomenon was that heterogeneous OBs were observed in some mature embryos, such as one LO (10QT0073: 39.16%), two HO (11DH623 and 14313: 50.68 and 52.75%), and three UO (TGY8, TGY10, and TGY14: 55.04, 58.57, and 59.86%). In these materials, most (over 95%) of OBs were smaller than 0.8 μm^2^, while some unusual OBs were several times bigger (some even over 20 μm^2^) than the average size of *B. napus* seed OBs (Figure 1; Table 1).

In all examined materials, the number of PSVs in each cell was between a few to dozens and varied widely. Similarly, the average size of PSVs in the different materials also changed greatly. Moreover, beside TGY18 (58.89%) which had much bigger average PSVs size (Table S3), the average size of PSVs in UO group was larger than of the other three groups (Figure 2D), but the difference was not significant. According to our calculations, the total value of OBs and PSVs coverage in different groups was all above 80%, which showed a little difference between the different groups.

The cell wall thickness of the different groups was also calculated. The results showed that the average cell wall thickness in LO group was greater than of the others, but showed no remarkable differences among the four groups (Figure 1, red arrow; Figure 2E). While a LO material (10QT0073, 39.16%) had much thicker cell wall than any other materials, with a cell wall thickness in 1.26 μm (Table S3). These results indicated that the decreased cell wall thickness might be a reason for the formation of the higher oil content.

### Correlation analysis for OBs morphology and oil content and FA compositions

As shown in Table 3, the average OB size was significantly negatively correlated with the protein and glucosinolates content with coefficients of –0.531 (*p* < 0.01) and −0.411 (*p* < 0.05), while was significantly positively correlated with oil content with a coefficient of 0.474 (*p* < 0.05). Though, there was no significant correlation between average OB size and FA compositions, the average OB size correlated in C18:2 and C18:3 with relatively high correlation coefficients of −0.316 and 0.31.

**Figure 3:**
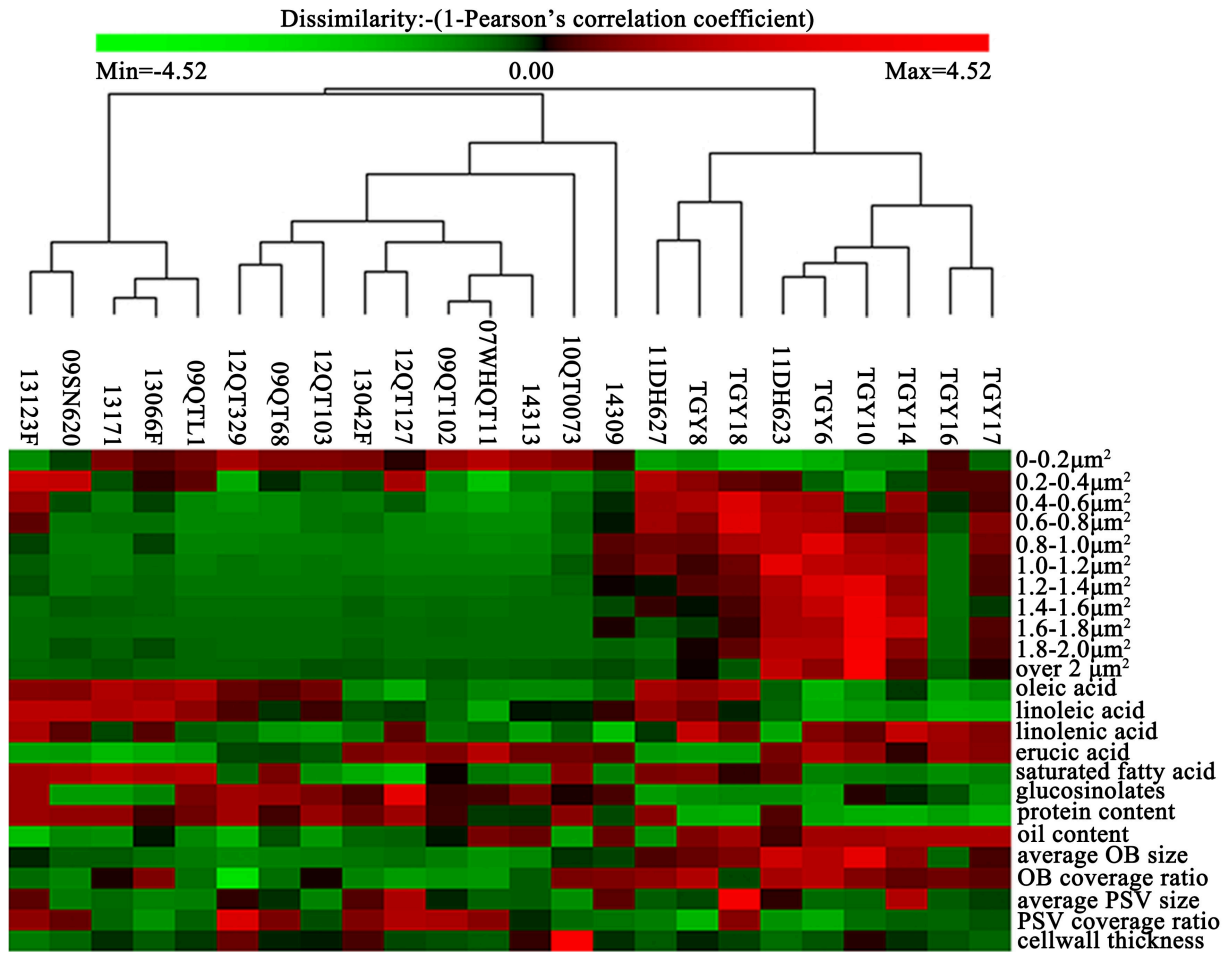
**Hierarchical cluster for the oil content, protein content, major FA compositions and the related morphology traits in the cotyledon cells of the 24 materials**. The data of the oil content, protein content, major FA compositions, and the related morphology traits in the embryonic cells of 24 materials were clustered (average linkage, correlation similarity).

In order to clearly understand the relationship between OBs morphology and oil content as well as FA compositions, the related traits of OB morphology were clustered based on Pearson correlation coefficient by the PermutMatrixEN (Figure 3). The results showed that the materials of UO group (i.e., TGY8, TGY10, TGY18, TGY6, TGY14, TGY17) were similar to each other in OBs size distribution except for TGY16 (Figure 3). Meanwhile, in other groups, the OBs size distribution of the materials in the same group was not always similar, and the materials in different group might have similar OBs size distribution, such as HO material 14313 (oil content of 52.75%) and LO material 10QT0073 (oil content of 39.16%) which were close to each other in the cluster tree (Figure 3). These results indicated that the OBs size distribution might be correlated with oil content. So we calculated the Pearson correlation coefficient between OBs distribution and oil content as well as FA compositions by SPSS 20.

As shown in Table 4, the proportion of OBs distributed in 0.6 to over 2 μm^2^ was significantly positively correlated with oil content. No significant correlation between the OBs smaller than 0.6 μm^2^ and oil content were observed. At the same time, the proportion of OBs distributed in 0–0.2 μm^2^ was significantly positively correlated with glucosinolates content with a coefficient of 0.459 (*p* < 0.05), while the proportion of OBs distributed in 0.4–1.2 μm^2^ were significantly negatively correlated with glucosinolates content (Table 4). Similarly, the proportion of smaller OBs was significantly positively correlated with C18:2, but the proportion of bigger OBs was significantly negatively correlated with C18:2. Except that, the proportion of OBs distributed in 0–0.6 μm^2^ correlated with C18:3 with coefficients of −0.5 (*p* < 0.01), 0.405 (*p* < 0.05), and 0.508 (*p* < 0.05), and the proportion of OBs distributed in 0.2–0.4 were also correlated with C22:1 with a coefficient of −0.544 (*p* < 0.01).

**Table 4:** **The correlation analysis for the distribution of OB size, oil content and FA compositions**.

**Distribution of OB size**	**Oil content**	**Oleic acid**	**Linoleic acid**	**Linolenic acid**	**Erucic acid**	**Saturated fatty acid**	**Glucosinolates**
0–0.2	−0.369	−0.111	0.042	−0.500^*^	0.18	−0.103	0.459^*^
0.2–0.4	−0.234	0.395	0.516^**^	0.405^*^	−0.544^**^	0.37	−0.09
0.4–0.6	0.351	0.22	−0.001	0.508^*^	−0.267	0.122	−0.472^*^
0.6–0.8	0.460^*^	0.099	−0.187	0.379	−0.101	0.018	−0.534^**^
0.8–1.0	0.585^**^	−0.147	−0.355	0.341	0.139	−0.111	−0.514^*^
1.0–1.2	0.489^*^	−0.187	−0.362	0.177	0.2	−0.088	−0.450^*^
1.2–1.4	0.566^**^	−0.287	−0.437^*^	0.294	0.282	−0.188	−0.365
1.4–1.6	0.514^*^	−0.263	−0.410^*^	0.255	0.269	−0.186	−0.307
1.6–1.8	0.570^**^	−0.3	−0.468^*^	0.278	0.307	−0.243	−0.247
1.8–2.0	0.530^**^	−0.274	−0.423^*^	0.238	0.284	−0.169	−0.29
over 2	0.419^*^	−0.316	−0.397	0.104	0.328	−0.173	−0.191

* Significant at p = 0.05;

** *Significant at p = 0.01*.

### Candidate genes confirmation by analyzing the QTL interval region for oil content

As mentioned above, the OB morphology was correlated with FA compositions and oil content in *B. napus*, and previous researches already indicated that the morphology of OB might have relation with the genes associated with OB-membrane proteins, cell progress (such as cell growth, cell division, cell proliferation, etc.), and TAG metabolism. To interpret the controlling mechanism for the OB morphology in *B. napus*, the candidate genes associated with OB-membrane proteins, cell progress, and lipid metabolism was performed in mapping analysis of the QTL interval region of oil content, protein content, and FA compositions.

**Figure 4:**
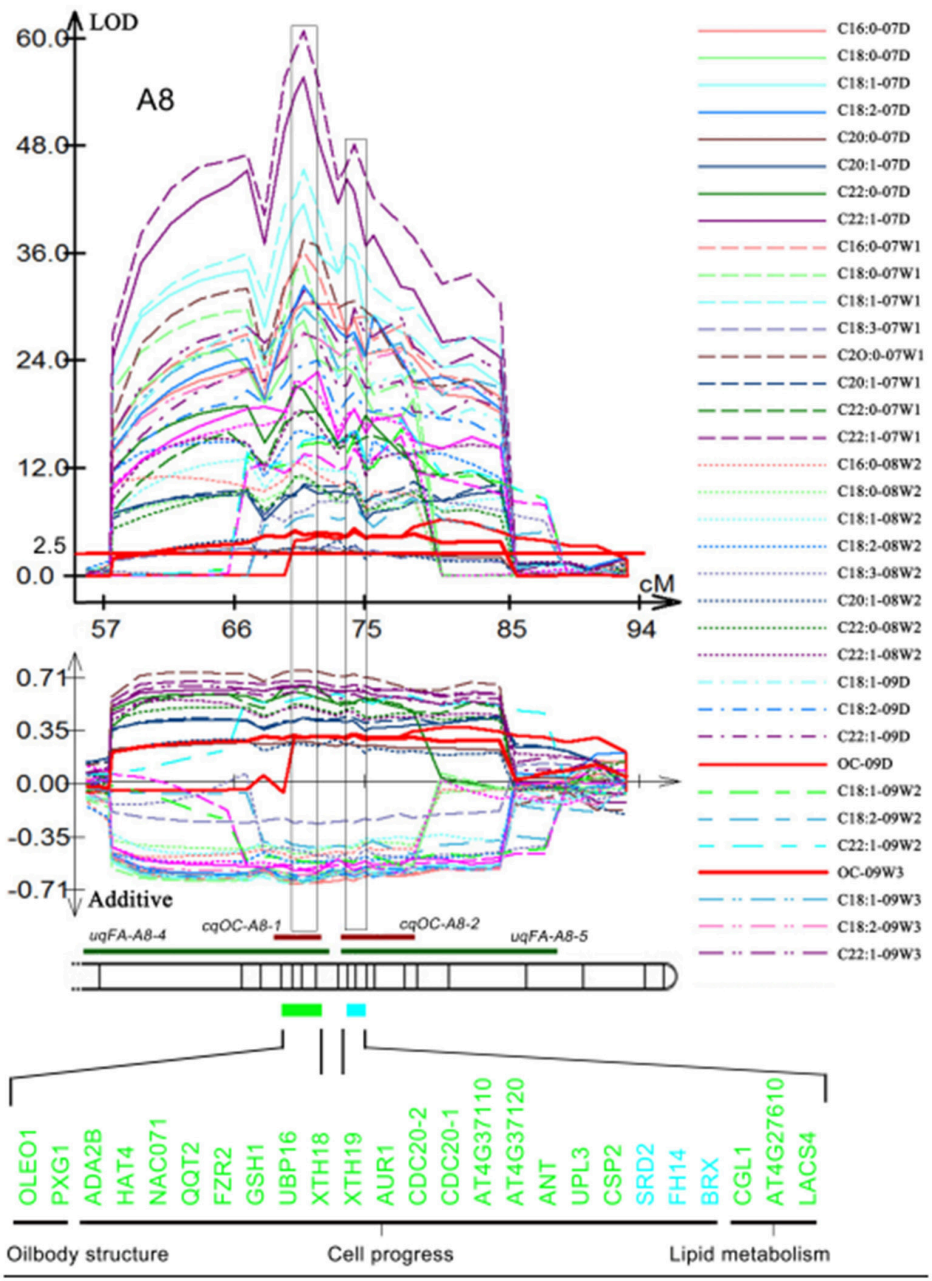
**Demonstration of co-localized QTLs of oil content and FA compositions in the different environments**. Original QTLs identified in the different experiments are showed by curves above the material of linkage group and their additive effect with one standard deviation are showed by curves of the same color below the material of the linkage group. Different kinds of materials represent different environments, which were shown on the right. The long red material in the LOD value of 2.5 was used for identifying statistically significant QTL. Then the chromosome bar (bands represent marker position) is used to exhibit co-localization of consensus QTL for oil content (red narrow bar) and unique QTL for FA (green narrow bar). The blocks aligned to confident intervals also displayed by light green rectangle (U block) and light blue rectangle (B block), in which 25 genes (from which block, they were marked using a corresponding color) associated with OB-membrane proteins, cell progress and lipid metabolism are listed.

A total of 63 individual QTLs associated with the content of ten different FAs were detected in TN population based on oil content and FAs which were obtained in the year of 2007, 2008, and 2009 (Gan et al., 2013; Wang et al., 2015). Fifteen and eighteen consensus QTLs were obtained for seed oil and protein content, respectively, and four QTLs were mapped in the same CI that controlled protein and oil content with opposite additive effect (Gan et al., 2013; Wang et al., 2015). Three and five of QTLs for FAs showed a close linkage with QTLs for seed oil and protein content, respectively (Table S4). Two hundred and one of the two hundred and seventy-one candidate genes were located in at least one QTL in the TN genetic map (Table S5). Among them, 94 candidate genes were located in two different QTLs, with 27 candidate genes, including two genes associated with OB-membrane proteins, four lipid metabolism-related genes, and 21 cell process related genes located in three different QTLs (Table S5). In the overlapped CIs of *uqFA-A8-5* and *cqOC-A8-1, uqFA-A8-5*, and *cqOC-A8-2*, 20 cell process-related genes, two OB-membrane-proteins-related genes and three lipid metabolism-related genes were observed (Figure 4). Moreover, six cell process-related genes, *CLV3* (*AT2G27250*), *AUR2* (*AT2G25880*), *CDC6* (*AT2G29680*), *GPA1* (*AT2G26300*), *PRS* (*AT2G28610*), *LHW* (*AT2G27230*), and one gene associated with OB-membrane proteins, *AT2G25890* were mapped on overlapped CI of *cqPC-A4-2*, and *uqFA-A4-3* (Table S5). Another map alignment analysis was also performed based on the genetic linkage map of KN population. One hundred and thirty-one candidate genes were located in at least one QTL (Table S5). Forty-three candidate genes were located in two different QTLs and seven candidate genes were located in three different QTLs. Ninety-eight candidate genes were co-identified in the genetic linkage map of both TN and KN population (Table S5).

**Figure 5:**
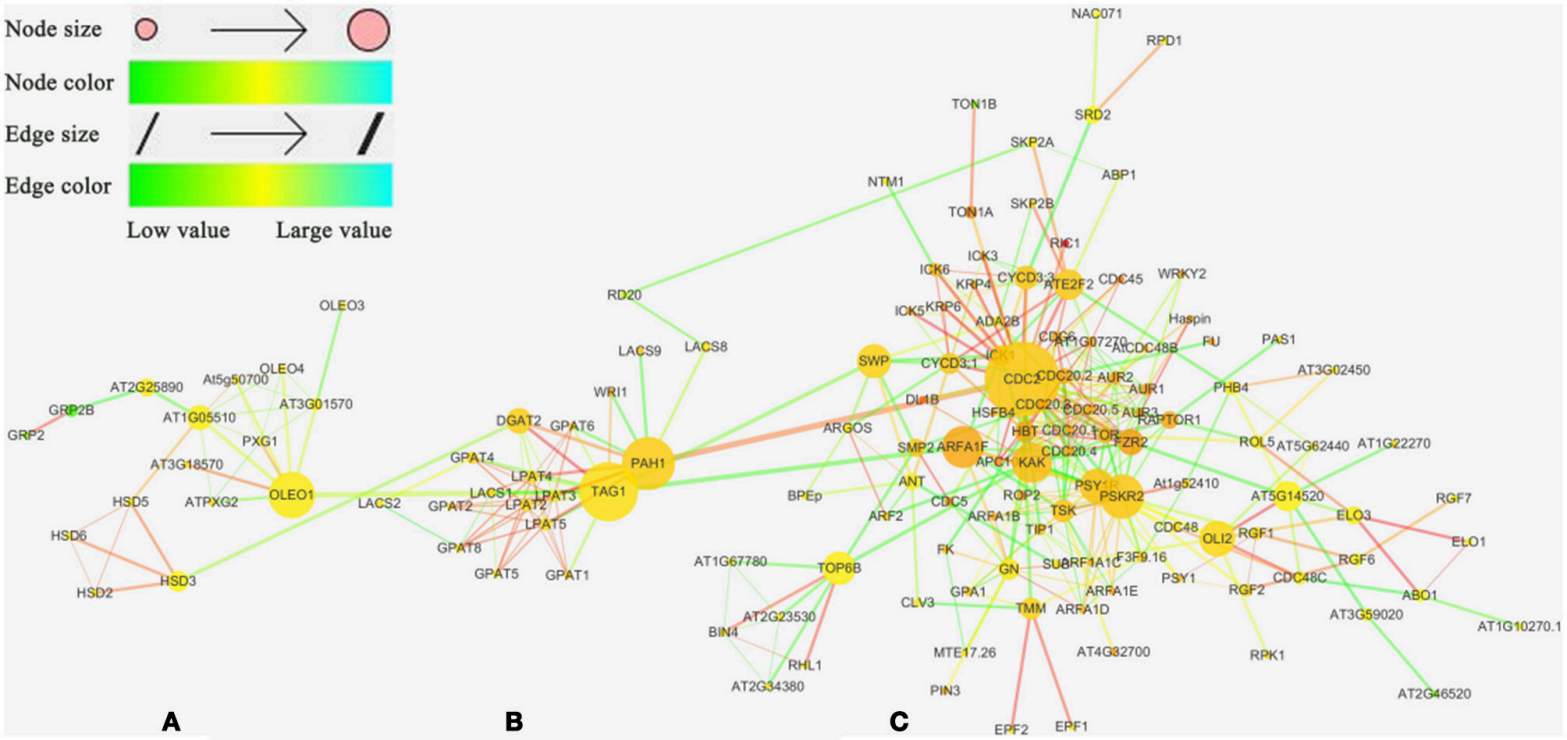
**The interaction network of candidate genes**. Network visualization for interaction of the 201 candidate genes observed from the QTL results using Cytoscape_V3.2.0 software. Genes are presented as nodes and gene interactions are presented as edges. **(A)** OB-membrane-related genes cluster. Different classification genes indicated by different shape with different color (red round rectangle, OB-membrane-related gene; yellow ellipse, TAG metabolism-related gene; blue diamond, cell process-related gene). **(B)** TAG metabolism-related genes cluster. **(C)** cell process-related genes cluster.

String was used to investigate the interaction of candidate genes identified in TN population genetic linkage map and the interaction network was constructed by Cytoscape_V3.2.0. The results revealed that the interaction network could be divided into three sub-clusters: OB-membrane-proteins-related genes cluster (A cluster; Figure 5A), TAG metabolism-related genes cluster (B cluster; Figure 5B) and cell process-related genes cluster (C cluster; Figure 5C). The A cluster was composed of 14 genes associated with OB-membrane-proteins and two cell progress-related genes, *GRP2* (*AT4G38680*), and *GRP2B* (*AT2G21060*), in which the genes were formed tightly in linked group. In this group, *HSD3* (*AT3G47360*, encoding a steroleosin) was directly affected by *DGAT2* (*AT3G51520*) and *OLEO1* was directly affected by *TAG1* (*AT2G19450*), the critical gene in the final step of TAG synthesis, while two cell progress-related genes were linked with *AT2G25890* (Oleosin-like protein). The B cluster consisted of one gene associated with OB-membrane proteins, *RD20* (*AT2G33380*) and 18 genes associated with TAG metabolism. *RD20* was affected by a lipid metabolism-related gene, *LACS8*, and a cell progress-related gene, *SKP2A* (*AT1G21410*) (Figure 5). *PAH1* (*AT3G09560*), which catalyzes the conversion of PA to DAG, was linked to an important cyclin-dependent kinase gene, *CDC2* (*AT3G48750*) (Gaamouche et al., 2010). *TAG1* was combined with two cell progress-related genes, *SWP* (*AT3G04740*, mediator of RNA polymerase II transcription subunit 14) and *ARFA1F* (*AT1G10630*, ADP-ribosylation factor A1F). The C was all the cell progress-related genes. Most of these genes were combined with others tightly and formed a complex interaction network.

The network mentioned above indicated that the genes involved in OB-membrane proteins, lipid metabolism and cell progress were directly or indirectly affected each other. These results showed that the morphology of OB was associated with the oil content and FA compositions at genetic level.

**Figure 6:**
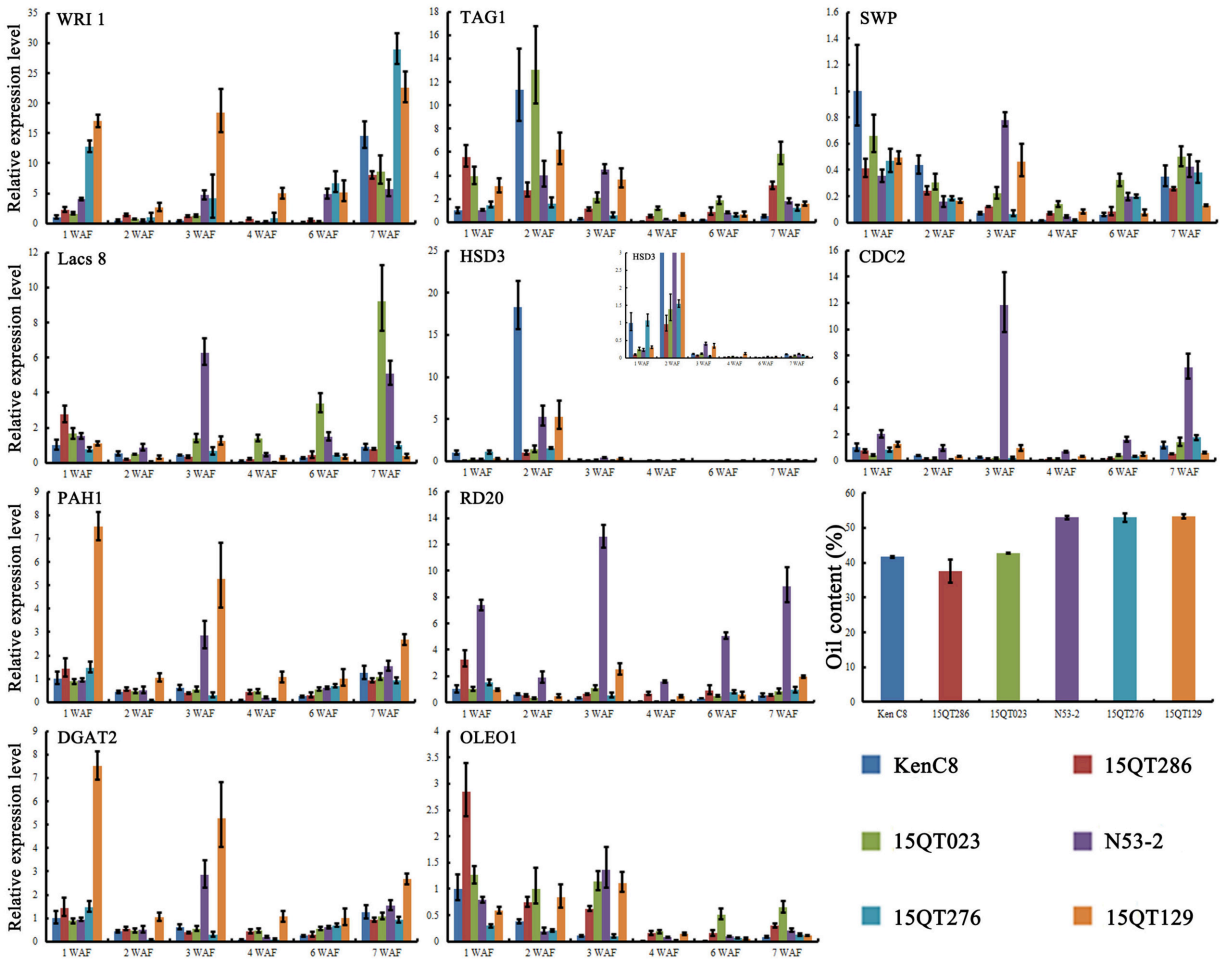
**The expression analysis for key candidate genes of the potential relation pathway in high and low oil content materials**. For each reaction, three technical replicates were conducted. Bar represent the standard error calculated by _ΔΔ_CT method.

The expression analysis for key candidate genes in high and low oil content materials except for *SKP2A*, 10 key candidate genes were selected for qRT-PCR analysis in order to further elucidate their role in the regulation of the morphology of OB (Figure 6). The developing seeds (1–4, 6, and 7 WAF) were used for the qRT-PCR analysis.

The differentially expression of six lipid metabolism related genes in developing seed with high and low oil content materials was indicated by qRT-PCR analysis and the results were showed in Figure 6, in which four of them show higher expression (*WRI, Lacs8, PAH1*, and *DGAT2*) in high oil content materials. Further analysis revealed that the expression of *WRI* was higher in all development in high oil content materials (Figure 6). *TAG1* was lower expressed in high oil content materials at most development stages except at 3 WAF. The expression of *HSD3* was higher only at 2 WAF and show higher expression in N53-2 and 15QT129 than that of in 15QT023 and 15QT286, but significant lower than that of in Ken C8. Two OB-membrane proteins genes, *RD20* (caleosin 3) and *OLEO1* (oleosin 1) showed a complex expression pattern among different materials during development (Figure 6). *OLEO1* showed significant higher expression only in low oil content materials at 1 and 2 WAF. Similarly, *SWP*, a cell progress-related gene, also showed clearly higher expression only in low oil content materials at 1 and 2 WAF. Another cell progress-related gene, *CDC2* showed higher expression in high oil content materials, especially in N53-2. In conclusion, 10 genes, *WRI, Lacs8, PAH1, DGAT2, TAG1, HSD3, RD20, OLEO1, SWP*, and *CDC2* (Figure 6), showed significant difference expression level in high and low oil content seeds during the seed development.

## Discussion

### The morphology of OBs correlated with oil content and FA compositions

OBs are relatively simple but very important organelles (Siloto et al., 2006). Lipid stored in OBs of oilseeds is the food reserves for germination and post-germination growth of the seedlings (Tzen et al., 1993). It was reported that the OB size was ranged from 0.5 to 2.5 μm in orthodox seeds (Tzen et al., 1993; Murphy, 2001; Mantese et al., 2006). The relationship of the OBs morphology with the oil content and the FA compositions is still not sufficiently studied, especially in *B. napus* materials with UO. Hu et al. (2009) compared the OBs morphology in several *B. napus* materials. The unusually large OBs (over 5.0 μm) in the LO rapeseed materials were observed, and they speculated that the unusually large OBs were highly correlated with the decrease of oil content in *B. napus*, but only five materials were investigated and the highest oil content of material contained only 49.7% of oil. They also studied the seed structure and the characteristics of different rapeseed oil content, including a UO rapeseed material (Hu et al., 2013). Their results showed that the high OB organelle to cotyledon cell area ratio and the cotyledon ratio in seeds were the main reasons for the formation of UO. They also indicated that the oil content was significantly negatively correlated with protein content, but was not correlated with FA compositions. While they did not pay any attention on the OB characteristics and its relationship with oil content and FA compositions, and the materials used in their research were also limited.

To investigate the OB characteristics in *B. napus* with UO and the relationship of OB morphology with the oil content and FA compositions, we compared the OBs morphology in the embryonic cells of 24 *B. napus* materials with oil content ranging from 34.54 to 60.61%. Our results indicated that the cross-sectional area of OBs were ranged from 0.01 to 2 μm^2^, and unusually large OBs (even over 20 μm^2^) were also observed in some rapeseed materials, and this was consistent with previous results (Murphy, 2001; Mantese et al., 2006; Hu et al., 2009, 2013). However, unusually large OBs were not present in all investigated LO materials, and also were observed in other MO, HO, and UO materials. That means the unusually large OBs were not highly correlated to the decrease of oil content in *B. napus*.

The average OBs size of different groups was also compared, and it was revealed that the average size of OBs in UO materials was clearly bigger than that in other three groups (Figure 2C). Though, the mechanism is still unclear, the correlation between the morphology of OBs and oil content already been identified by previous researches (Hu et al., 2009; Miquel et al., 2014). The positive correlation of glucosinolate content with oil, erucic acid, and total unsaturated fatty acid contents in *Eruca sativa* has already been reported by Sukhija et al. (1985). So it is no surprising that the Pearson correlation coefficient analysis showed that the average OB size was significantly negatively correlated with the protein and glucosinolates content, but it was significantly positively correlated with the oil content (Table 3). Consistent with that, the proportion of bigger OBs indicated that it was significantly negatively correlated with glucosinolates and C18:2 and significantly positively correlated with oil content. Meanwhile, the proportion of smaller OBs was significantly positively correlated with glucosinolates and C18:2. These results were similar to the observation in two inbred maize materials with high and low oil content. The HO material had a larger OBs and a higher TAG-to-oleosin ratio compared with the LO material (Ting et al., 1996). The current results showed that the size and stability of OB in a plant species might be highly depend on the ratio of oil to oleosin (Ting et al., 1996; Peng et al., 2003) and the small OBs could form larger ones by fusion or coalescence of each other during the formation (Miquel et al., 2014). So the formation of bigger OBs in UO materials may be due to the UO that might increase in the ratio of oil to the oleosins and force the OBs to be close to each other and fuse.

### The morphology of OBs associated with oil content, FA compositions, and cell progress by complex gene interaction network

**Figure 7:**
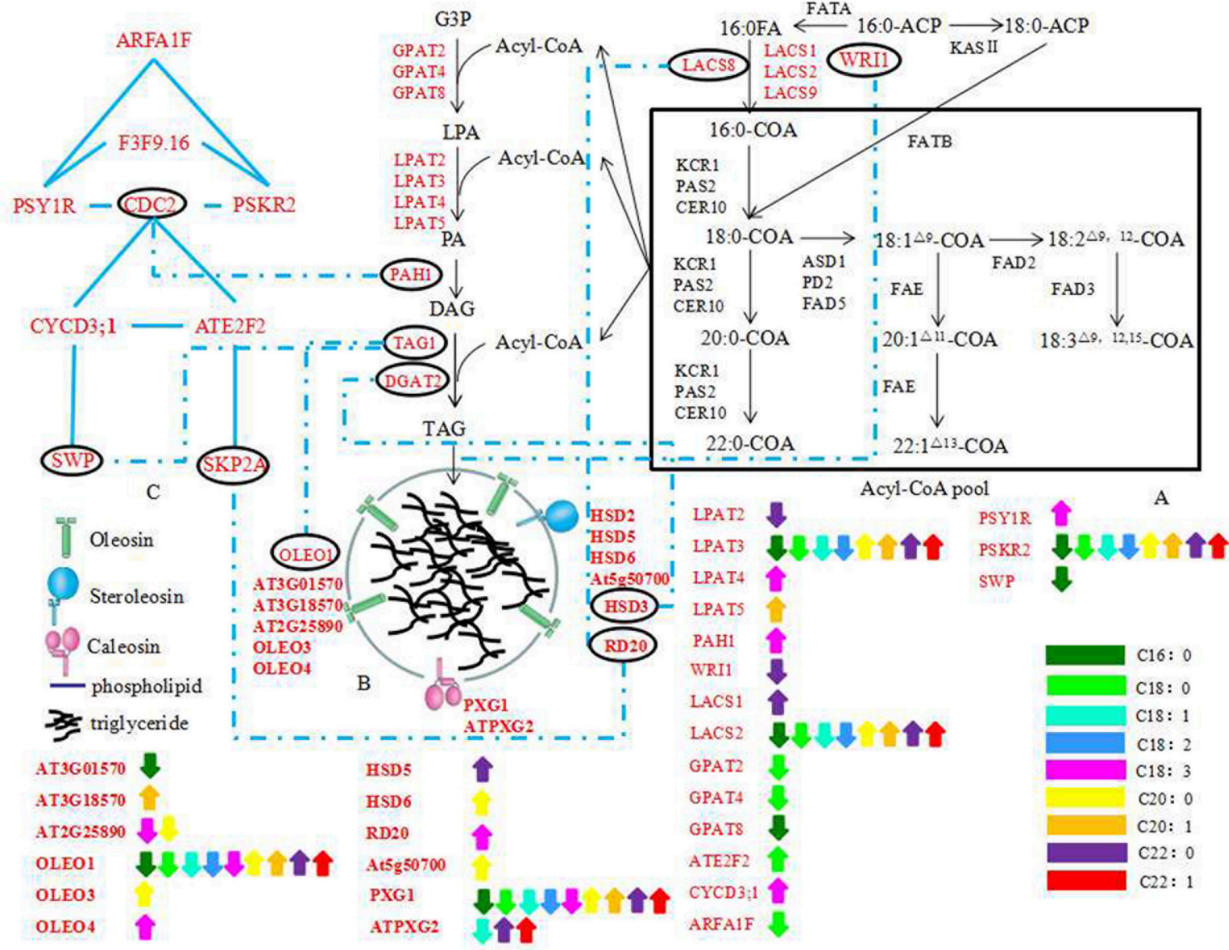
**The potential regulation pathway of the genes associated with OB morphology, oil content and FA compositions. (A)** FAs biosynthetic and Acyl-CoA pool formation; **(B)** a schematic model of mature oil body surrounded by OB-membrane proteins; **(C)** a simple Schematic Model of cell division, growth, proliferation. The black characters indicate genes detected in previous studies, the red characters indicate the candidate genes in this study. The blue dotted materials indicate the combine between genes in different classification. The Blue materials indicate the combine between genes in same classification. The double-headed arrows indicate genes underlying different QTLs which affect the same trait with opposite additive effect at the same time based on the WinQTLcart2.5 and QTLNetwork_2.0 results (not the combined results of the two). Different colors denote different traits as the bar showed at the lower right corner of the picture. The abbreviations of gene name are same with Table S5.

On the basis of QTLs mapping, we found 198 and 131 genes located in the CIs of QTLs for oil content, protein content, and FA compositions in TN population and KN population genetic linkage map, respectively. Then a potential pathway for the interaction of the OB morphology, oil content, and FA compositions in *B. napus* was constructed based on the analysis of the 198 candidate genes found in the TN population genetic linkage map. The formation of OB was a highly coordinated process that involves carbon metabolism, FA synthesis, TAG synthesis pathways, and even cell differentiation pathways (Cheng et al., 2009; Yang et al., 2012; Thiam et al., 2013; Miquel et al., 2014). Consistent with that, many genes associated with OB membrane-related proteins, TAG synthesis, and cell progress regulatory pathway were included in the potential pathway (Figure 7).

Oleosins, caleosins, and steroleosins are the three major OB-membrane protein families (Shimada and Hara-Nishimura, 2010). Miquel et al. (2014) monitored the dynamics of OBs in living *Arabidopsis* embryos at different stages of development in the wild type and 3 mutants affected in the accumulation of various oleosins (*OLE1, OLE2*, and *OLE4*). In single, double, and triple mutant backgrounds, the size and spatial distribution of OBs were modified, affecting in turn the total lipid content, which suggests that the oleosins have specific roles in the dynamics of OBs during seed development. The results revealed the relationship between the morphology of OBs and oleosins. Some more previous findings also indicated that the size and stability of OB in a plant species might be highly depend on the ratio of oil to oleosins (Ting et al., 1996; Peng et al., 2003). Twenty-three genes associated with OB-membrane proteins were found possibly correlated with oil content, protein content or FA compositions (Table S5). These genes formulated a tightly network and some genes interacted with genes associated with TAG synthesis or cell progress regulatory pathway, including *OLEO1, HSD3* (encode a steroleosin protein), and *RD20* (encode a steroleosin protein) (Figures 5, 7). These results indicated that the OB-membrane proteins might have important roles in controlling OB morphology and the relationship of OB morphology with the oil content, protein content and FA compositions.

OB fusions and fissions, organelle interactions, or transport cannot simply be explained by the function of OB-membrane proteins (Walther and Farese, 2009; Thiam et al., 2013). In animal, net transfer of storage lipids from smaller LDs to larger ones without redistribution of LD proteins from donor to acceptor was shown (Gong et al., 2011). And the genes involved in TAG synthesis might re-localize to grow LDs and mediated LDs growth (Wilfling et al., 2013). Recently, it was also confirmed that over-expression of *BnDGAT2*, an important enzyme of TAG synthesis, could significantly alter the FAs profile and enhance the oil production in green microalga (Ahmad et al., 2015). These results indicated a growth process independent of fusions with existing LDs or with the ER possibly present in plants (Wilfling et al., 2013). Here, we found 25 genes associated with TAG synthesis located in CIs of oil content, protein content or FAs QTLs by map alignment analysis and these genes might directly or indirectly interact with the genes associated with OB-membrane proteins (Table S5; Figures 5, 7). So even there was still no TAG synthesis-related protein founded in the surface of *B. napus* OBs (Katavic et al., 2006), we do not allow the elimination of possibilities of the existence of the growth process independent of fusions with existing OBs or with the ER.

The difference in the mechanisms of OBs formation may depend on the type of OB, on cell type and on the neutral lipids accumulated (Cheng et al., 2009; Thiam et al., 2013). Moreover, studies in mesocarp from avocado (*Persea americana*) and olive (*Olea europaea*) fruits revealed that oleaginous fruit cells contained larger OBs than seeds and oleosins were not present in fruit OBs (Ross et al., 1993). Liu et al. (2012) enhanced the seed oil production by over-expressing a transcription factor, *BnGRF2*, while some genes which were up-regulated in the transgenic *Arabidopsis* were classified as being associated with cell proliferation, oil synthesis and storage processes, such as *KCS16, GPAT*, and oleosin proteins. These results indicated that the genes associated with cell progress regulatory pathway (cell growth, cell division, cell proliferation, and cell morphogenesis) might have specific roles in the change of oil content and the morphology of OBs. In this paper, we found 21 cell progress regulatory pathway-related genes located in the CIs of QTLs for protein, oil content, and FAs at the same time (Table S5). Cell progress was controlled by a complex regulatory network (Gaamouche et al., 2010; Figure 5). Some genes, such as *CDC2, CYCD3;1, SKP2A*, and *SWP*, were not only directly or indirectly linked with TAG synthesis related genes but also interacted with the genes associated with OB-membrane proteins. These results indicated that the genes associated with cell progress regulatory pathway might alter the morphology of OBs as well as the oil content and lipid compositions by direct or indirect interaction with the genes associated with OB-membrane proteins and TAG synthesis.

In order to assess the feasibility of this potential regulation pathway, the expression of 10 key candidate genes in the pathway was compared in the developing *B. napus* seeds with high and low oil content by qRT-PCR. It was indicated that all of these genes had significant difference in expression in the developing high and low oil content *B. napus* seeds (Figure 6). Such as *WRI*, a master transcription factor regulating seed oil biosynthesis, showed higher expression in high oil content material during development (Figure 6; Kanai et al., 2015). In *Arabidopsis*, the extension of *WRI1* expression during mid-phase of seed development significantly enhanced seed oil content and the morphology of OBs showed significant change at the same time (Kanai et al., 2015). These results imply that these key candidate gene maybe do participate the regulation of the morphology of OBs during the *B. napus* seed development.

To conclude, we indicated that the morphology of OBs was correlated with oil content and FA compositions by microstructure comparison of OBs in *B. napus* with various oil content and found many genes associated with TAG synthesis, OB-membrane proteins, and cell progress regulatory pathway co-located in the overlapped CIs of QTLs for oil content, FA compositions and protein content by QTLs mapping. Moreover, these genes associated with TAG synthesis and cell progress regulatory pathway might directly or indirectly affect the genes associated with OB-membrane proteins. Our results suggest that the morphology of OBs might be directly controlled by the genes associated with OB-membrane proteins, and the genes associated with TAG synthesis and cell progress regulatory pathway might affect the OBs morphology through the gene-gene interaction with the genes associated with OB-membrane proteins. This could partially explain why the morphology of OBs was associated with oil content and FA compositions.

## Authors contributions

JG carried out the microstructure comparison, data analysis, qRT-PCR, and wrote the manuscript. HC performed the map alignment work. HW, YHL, DL, JX, JG, GL, XZ, and YL participated in the field experiment. ML designed, led, and coordinated the overall study.

### Conflict of interest statement

The authors declare that the research was conducted in the absence of any commercial or financial relationships that could be construed as a potential conflict of interest.
